# Photophysics of Azobenzene Constrained in a UiO Metal–Organic Framework: Effects of Pressure, Solvation and Dynamic Disorder

**DOI:** 10.1002/chem.202101879

**Published:** 2021-10-04

**Authors:** Alif Sussardi, Ross J. Marshall, Stephen A. Moggach, Anita C. Jones, Ross S. Forgan

**Affiliations:** ^1^ EaStCHEM School of Chemistry The University of Edinburgh David Brewster Road Edinburgh EH9 3FJ UK; ^2^ WestCHEM School of Chemistry University of Glasgow University Avenue Glasgow G12 8QQ UK; ^3^ School of Molecular Sciences The University of Western Australia 35 Stirling Highway Perth Western Australia 6009 Australia

**Keywords:** metal–organic frameworks, azobenzene, high-pressure chemistry, fluorescence, X-ray diffraction

## Abstract

Photophysical studies of chromophoric linkers in metal–organic frameworks (MOFs) are undertaken commonly in the context of sensing applications, in search of readily observable changes of optical properties in response to external stimuli. The advantages of the MOF construct as a platform for investigating fundamental photophysical behaviour have been somewhat overlooked. The linker framework offers a unique environment in which the chromophore is geometrically constrained and its structure can be determined crystallographically, but it exists in spatial isolation, unperturbed by inter‐chromophore interactions. Furthermore, high‐pressure studies enable the photophysical consequences of controlled, incremental changes in local environment or conformation to be observed and correlated with structural data. This approach is demonstrated in the present study of the *trans*‐azobenzene chromophore, constrained in the form of the 4,4’‐azobenzenedicarboxylate (abdc) linker, in a UiO topology framework. Previously unobserved effects of pressure‐induced solvation and conformational distortion on the lowest energy, nπ* transition are reported, and interpreted the light of crystallographic data. It was found that *trans*‐azobenzene remains non‐fluorescent (with a quantum yield less than 10^−4^) despite the prevention of *trans‐cis* isomerization by the constraining MOF structure. We propose that efficient non‐radiative decay is mediated by the local, pedal‐like twisting of the azo group that is evident as dynamic disorder in the crystal structure.

## Graphical abstract

**Putting the pressure on azobenzene photophysics**: When locked in a UiO metal–organic framework, the azobenzene chromophore is geometrically constrained but exists in spatial isolation, unperturbed by inter‐chromophore interactions. This is a unique and advantageous environment in which to explore fundamental photophysical properties. Optical spectroscopy in a diamond anvil cell enables the photophysical consequences of pressure‐induced changes in solvation and conformation of the chromophore to be observed and correlated with crystallographic data.

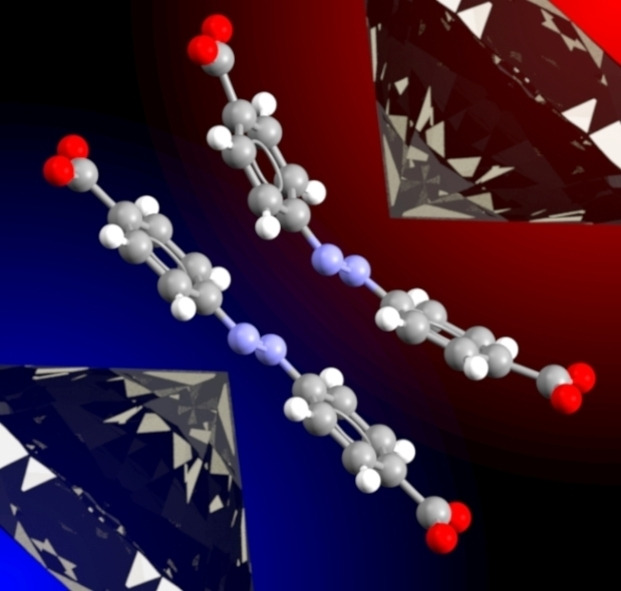

## Introduction

Metal–organic frameworks (MOFs), materials where metal ions or clusters are bridged by organic linkers into multidimensional networks,^[1]^ have long been proposed as potential luminescent sensors as a consequence of their high molecular storage capacities and ease of functionalisation.^[2]^ Chromophores can be installed at metal clusters or by using intrinsically luminescent organic linkers, and changes in their emission on exposure to a range of differing analytes are the basis of their application in sensing. Mechanistic information in the form of electronic or structural changes on binding analytes is not often provided when new sensors are reported, despite the associated opportunities for both device optimisation and acquisition of fundamental knowledge.^[3]^ Network solids such as MOFs offer a unique environment to study chromophores; we have previously demonstrated that pressure‐induced changes in emission spectra of a Hf(IV) MOF with 1,4‐phenylene‐bis(4‐ethynylbenzoate) linkers can be directly correlated with dynamic linker rotation through associated single crystal diffraction and absorbance/emission spectroscopies.^[4]^ When a chromophore is incorporated as a linker in a MOF, its photophysical properties may be influenced by geometric restrictions imposed by the framework structure or through the introduction of interacting molecules into the pores. Generally, such effects are studied in the context of sensing applications, in the search for readily observable changes of optical properties of the MOF in response to external stimuli.^[2]^ However, the MOF construct also provides a unique and advantageous environment in which to explore fundamental photophysical properties. The chromophore is geometrically constrained and its structure can be determined crystallographically, but it exists in spatial isolation, unperturbed by inter‐chromophore interactions.^[5]^ Moreover, the controlled application of pressure to the framework allows the effect of incremental changes in conformation to be observed.^[4]^

**Figure 1:**
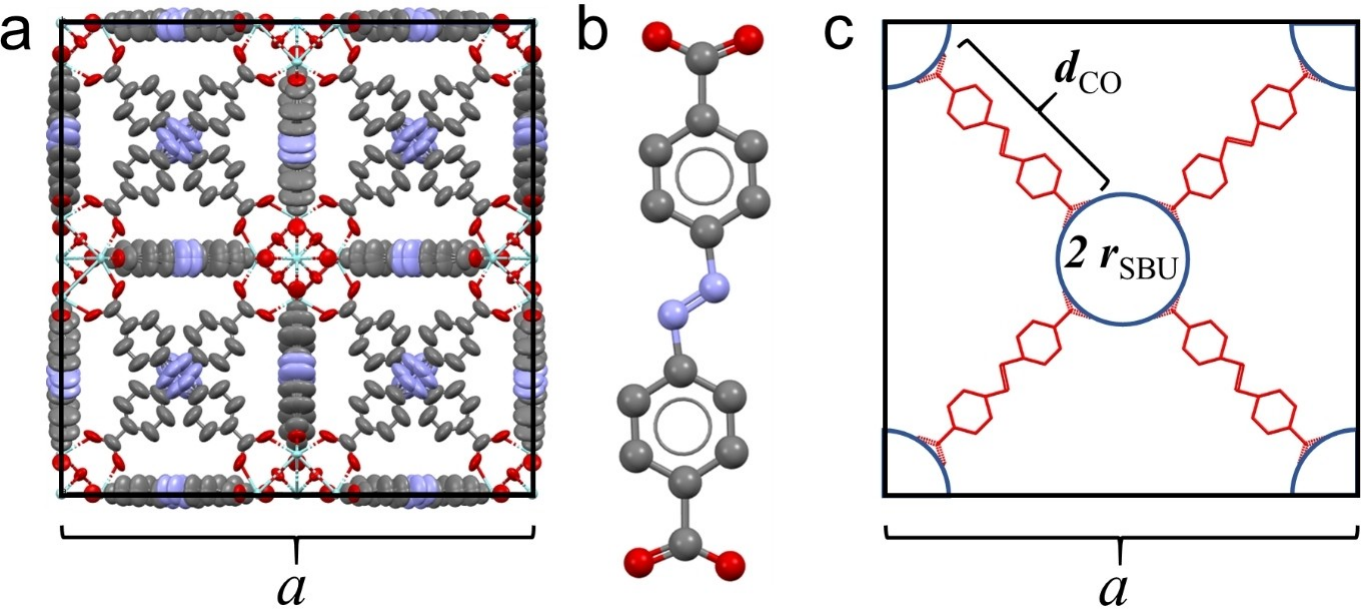
(a) The UiO‐abdc unit cell, (b) the structure of the azobenzene dicarboxylate (abdc) ligand and (c) a schematic diagram showing the ligand length, *d*_CO_, the radius of the secondary building unit (SBU), r_SBU_, and the unit cell parameter, *a*. These are related by: $a\sqrt{2}=4\;r_{SBU}+2\;d_{CO}$ .

We report here a study of pressure‐induced changes in the photophysics of the azobenzene chromophore, in the form of the 4,4’‐azobenzenedicarboxylate (abdc) linker, in the UiO‐abdc MOF, [Zr_6_O_4_(OH)_4_(abdc)_6_]_n_, as shown in Figure 1. Previously, we have reported the crystal structure of UiO‐abdc and the effect of high pressure on its unit‐cell volume.^[6]^ In a non‐penetrating hydrostatic medium, Fluorinert FC‐70, a large decrease in unit‐cell volume (almost 10 %) occurred on increasing the pressure to 1.8 GPa, while remaining crystalline. In contrast, in a pore‐penetrating hydrostatic liquid, methanol (MeOH), the UiO‐abdc framework proved to be exceptionally incompressible up to 4.8 GPa. In the present study, we explore the photophysical effects of pressure in these two different regimes, revealing both conformational and solvation effects on the energy of the nπ* transition.

## Results and Discussion

Azobenzene is the archetypal photo‐switchable chromophore. The large decrease in molecular length, 3.5 Å, produced by *trans* to *cis* photoisomerization is exploited extensively in photoresponsive biosystems, photopharmacology, smart materials and molecular machines.^[7]^ Azobenzene is also remarkable for its extremely low fluorescence quantum yield, 10^−6^ to 10^−7^ in solution,^[8]^ the consequence of the rapid (sub‐ps) internal conversion process that is intrinsic to the photoisomerization mechanism. Excitation to the Franck‐Condon geometry is followed by relaxation to a conical intersection between the excited (S_1_) and ground‐state potential energy surfaces. At the conical intersection, rapid internal conversion to the ground state occurs, followed by relaxation on the ground state surface to either the *cis* or *trans* equilibrium geometry. In spite of decades of intensive study, the precise details of the isomerization mechanism, such as the geometry at the conical intersection between excited and ground states, and the relaxation pathway on the excited state surface, remain a matter of debate. For excitation to the S_1_ (nπ*) state, experimental and computational studies of the isolated molecule increasingly point to a complex, multidimensional relaxation pathway involving both C−N=N−C torsion and N=N−C inversion.^[9]^ The persistence of rapid photoisomerization in viscous media or conformationally restricted systems has been explained in terms of a hula‐twist mechanism, in which switching is governed by a localised, pedal‐like motion (coupled single‐ and double‐bond rotation) of the C−N=N−C group in the first excited state, with minimal motion of the phenyl rings.^[10]^

One aspect of the study of the photophysics and photochemistry of azobenzene has been the quest for systems in which structural change is inhibited by the local molecular environment. If isomerization could be switched off, it might be expected that fluorescence would be switched on. In crystals of *trans*‐azobenzene, photoisomerization is prevented in the bulk of the lattice, although it has been observed for molecules at the crystal surface.^[11]^ In spite of the absence of isomerization, fluorescence does not appear to have been detected for macroscopic crystals. However, fluorescence has been reported for a crystalline nanowire of *trans*‐azobenzene.^[12]^ A fluorescence lifetime of 1.17 ns was measured, corresponding to a fluorescence quantum yield of about 10^−3^ (based on a radiative lifetime of ∼600 ns),^[8]^ but the crystal structure of the nanowire could not be determined, and the emission may have arisen from azobenzene aggregates rather than the individual chromophore. Bahrenburg et al.^[13]^ reported that the fluorescence quantum yield of 4,4’‐bis(acetamido)‐azobenzene was increased by a factor of 20 when it was cross‐linked, by a covalent bond at each end, into a poly(butylmethacrylate) polymer network. Nevertheless, the quantum yield remained low, at only 5 x 10^−3^, indicating efficient non‐radiative decay, despite the restriction of molecular motion in the tight polymer network. A high‐pressure study of an azobenzene‐functionalised polymer (poly(disperse red 1 acrylate)) found that photoisomerization was undetectable at pressures above 1.5 GPa, but fluorescence measurements were not made.^[14]^ In the UiO‐abdc MOF investigated here, both ends of the azobenzene chromophore are anchored to the framework (Figure 1), preventing the large decrease in length required to accommodate *trans*‐*cis* isomerisation, yet we were unable to detect any fluorescence by conventional spectrophotometry, and estimate the quantum yield to be <10^−4^. Informed by our previous crystallographic study,^[6]^ we are able to relate the efficient non‐radiative decay to dynamic disorder of the azo moiety.

**Figure 2:**
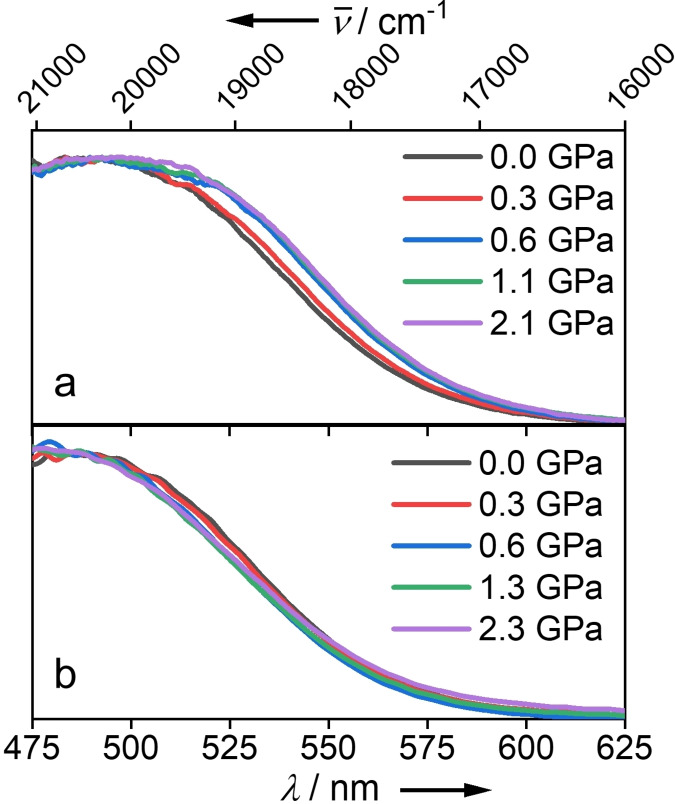
The normalised UV‐vis absorption spectra of UiO‐abdc as a function of pressure_,_ with (a) Fluorinert FC‐70 and (b) methanol as the hydrostatic medium.

The UV‐vis absorption spectra of UiO‐abdc were measured as a function of pressure, as described previously,^[4]^ using two alternative hydrostatic media: methanol, which is able to enter the pores of the MOF structure, and Fluorinert FC‐70, which is non‐penetrating (see Supporting Information for experimental details). The spectra, presented in Figure 2, show the red edge of the lowest energy, nπ* transition of the azobenzene chromophore. This transition, which is symmetry‐forbidden for the *trans* isomer, appears only weakly in solution‐phase measurements, but is easily observed in the MOF crystal because of the high concentration of the azobenzene chromophore.

As can be seen in Figure 2 and Tables S1 and S2, changing the hydrostatic medium from FC‐70 to methanol causes a hypsochromic shift (450 cm^−1^) of the absorption band. This is due to solvation of the chromophore by the pore‐penetrating methanol. In non‐penetrating FC‐70, the pores are free from solvent (apart from possible traces of acetone remaining after drying). This so‐called negative solvatochromism, i.e, stabilization of the ground state relative to the excited state by polar solvation, is a characteristic of nπ* transitions. This effect has been widely studied for ketones (the C=O chromophore)^[15]^ and has been used as a test for computational models of solvation,^[16]^ but is rarely discussed in the context of azobenzenes. For the C=O chromophore, the blue shift can be explained in terms of electrostatic interaction of the polar solvent with the C=O dipole moment; with increasing solvent polarity, there is increasing stabilisation of the dipolar ground‐state relative to the Franck‐Condon excited state.^[15b]^ However, this argument does not apply to *trans* azobenzene, since it has no dipole. The blue shift observed here can be attributed to specific H‐bonding interaction of the methanol OH with the azo nitrogen lone pair.^[17]^ The nπ* transition results in a decrease in hydrogen‐bond strength in the excited state, and consequent increase in vertical excitation energy relative to the unsolvated chromophore. The blue shift of 450 cm^−1^ compares with an expected ground‐state hydrogen bond energy of around 2000 cm^−1^,^[18]^ suggesting that significant hydrogen bonding interaction between the azo group and methanol persists in the excited state.

**Figure 3:**
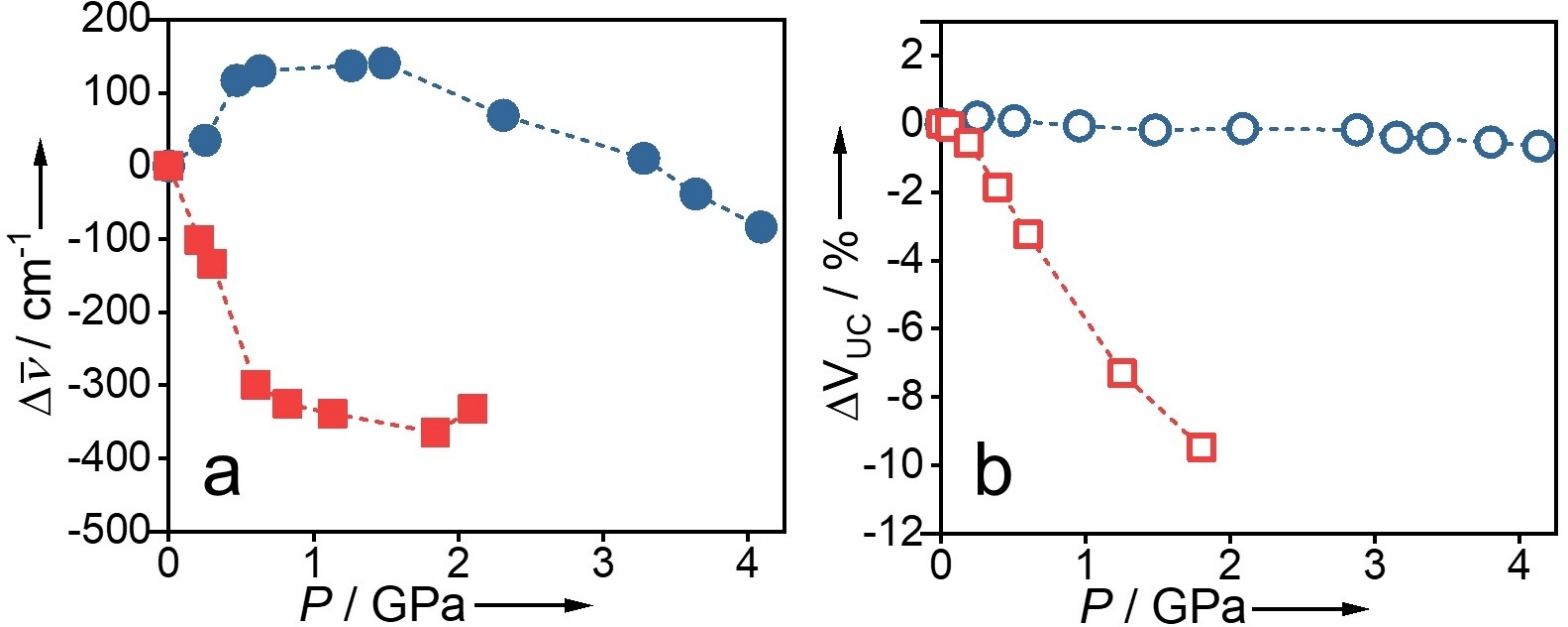
(a) Comparison of the pressure‐induced shift in wavenumber of the absorption red‐edge in methanol (blue circles) and in FC‐70 (red squares). (b) The pressure‐induced change in unit cell volume in methanol (blue open squares) and in FC‐70 (red open squares), using data from Ref. [6].

The pressure‐dependence of the absorption wavelength is quite different for the two pressure‐transmitting media, as shown in Figures 2 and 3 (a). In methanol, there is an initial hypsochromic shift with increasing pressure, which can be attributed to increasing solvation of the azo chromophore with increasing penetration of methanol. As the pressure is increased above 2 GPa there is a slight bathochromic shift with increasing pressure. In FC‐70, in contrast, there is a sizeable bathochromic shift as the pressure is increased to 1 GPa, with a levelling off above that pressure. This difference in the pressure‐response of the optical properties can be related to the previously reported difference in mechanical properties of the MOF framework in the two hydrostatic media.^[6]^ As illustrated in Figure 3 (b), penetration of methanol into the MOF framework results in a highly incompressible structure, with the unit cell volume decreasing by less than 1 % up to 4 GPa, whereas in non‐penetrating FC‐70 the unit cell volume decreases steeply with increasing pressure. We infer, therefore, from the correlation between spectroscopic and diffraction data, that the pressure‐induced bathochromic shift in the latter case is due to conformational change in the azobenzene chromophore caused by the enforced decrease in length of the linker. To test this hypothesis, we carried out DFT calculations to predict the pressure‐induced geometry change and TDDFT calculations to predict the consequent effect on the energy of the nπ* transition. The influence of conformational distortion on the energy of the nπ* transition is of current interest in the context of designing azobenzene derivatives with red‐shifted absorption spectra for biomedical applications.^[7c]^

**Figure 4:**
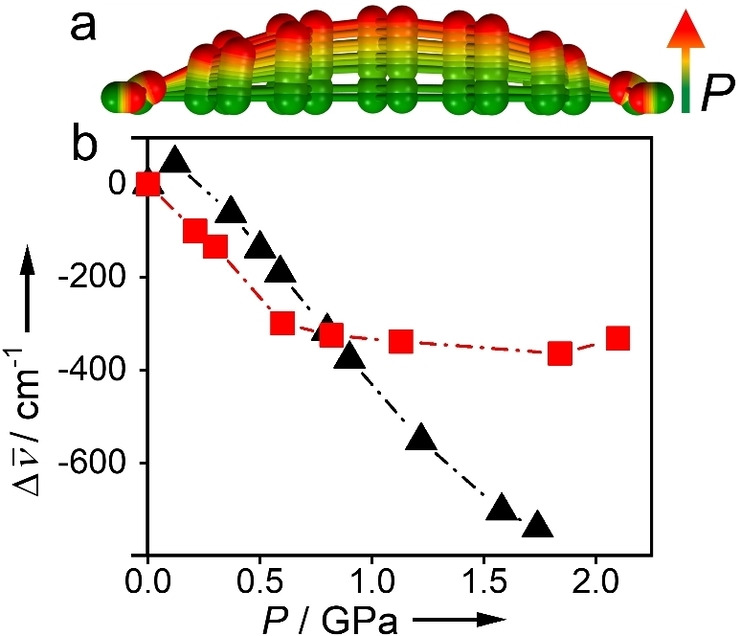
(a) The optimised geometry of abdc (with fixed *d*_CO_) as a function of pressure. (b) The measured bathochromic shift of the absorption spectrum red‐edge with increasing pressure in FC‐70 (red squares) in comparison with the TDDFT‐predicted shift (black triangles).

The length of the azobenzene dicarboxylate ligand (inter‐carboxylate distance, *d*_CO_) can be calculated from the unit cell parameters reported previously,^[6]^ as illustrated in Figure 1(c), and hence the dependence of *d*_CO_ on pressure determined (Figure S3). The values of *d*_CO_ used in the DFT calculations and the corresponding pressures are given in Supporting Table S3. Ground‐state geometries were predicted using the BHandHLYP level of theory and the 6–311 g(d,p) basis set, within Gaussian 09. Starting structures were based on the atomic‐resolution MOF crystal structure reported previously,^[6]^ where *d*_CO_ is 12.960 Å and the unit cell parameter *a*=29.3248 Å. The *d*_CO_ distance was fixed during the optimisation of the molecular geometry, and energy was minimised with respect to all other coordinates (as confirmed by frequency calculations). As shown in Figure 4(a), with increasing pressure the molecule becomes increasingly bent out‐of‐plane. The *trans* conformation about the azo group is maintained, but the phenyl rings are no longer coplanar (Supporting Figure S4). The vertical electronic transition energies were calculated for the optimised ground‐state geometries, using TDDFT with the same level of theory and basis sets, including transitions to 10 excited states. The predicted wavelengths of the first two electronic transitions, as a function of pressure, are given in Table S4. The lowest energy transition is confirmed to be nπ* in nature by examining the participating molecular orbitals (Figure S5) and shows characteristically low oscillator strength (Table S4). The nπ* transition is predicted to shift to lower energy with increasing pressure. As shown in Figure 4(b), the predicted magnitude of the bathochromic shift and the trend with increasing pressure are in excellent agreement with the experimental data, up to a pressure of ∼1 GPa. However, at higher pressures the observed and predicted trends deviate significantly; the observed absorption wavelength becomes essentially constant. This suggests that there is no further bending of the linker structure above 1 GPa, although the unit cell volume continues to decrease with increasing pressure according to our previously collected diffraction data (Figure 3(b)). Bending of the ligand is only one of the possible mechanisms by which the structure could compress. Shortening of Zr−O bonds in the Zr_6_O_4_(OH)_4_ core could also facilitate compression. We could even be seeing Zr−O bond breakage, as observed in previous pressure studies on UiO‐66 (with an even shorter terephthalic acid linker) under similar conditions.^[19]^ These effects would arrest any further distortion of the abdc linker and the associated bathochromic shift in absorption wavelength.

The pressure‐induced geometry change is also predicted to be accompanied by an increase in oscillator strength of the nπ* transition (Table S4). The bending distortion resembles a normal mode of a_u_ symmetry (ν_43_) that is active in vibronic coupling of the S_1_ and S_2_ transitions.[^9c^, ^20^] We are not able to detect the increase in oscillator strength quantitatively because of the saturation of the measured absorbance; however, it is visible as a change in colour of the crystal from yellow to increasingly intense red with increasing pressure, as the absorption in the blue/green becomes stronger.

The small bathochromic shift observed in methanol at the highest pressures is consistent with the same structural distortion. The shift produced at 4.1 GPa in methanol is equivalent to that at 0.19 GPa in FC‐70 (Figure 3(a)), and there is a corresponding equivalence in the respective unit cell volumes at these pressures. (Figure 3(b)). We also note that the slight increase in unit cell volume on initial compression in methanol^[6]^ may contribute to the hypsochromic shift observed at the lowest pressure (0.25 GPa).

We attempted to measure fluorescence from UiO‐abdc at ambient and high pressures, in both hydrostatic media, but the intensity was below our detection limit (see Supporting Information for experimental details). To estimate the fluorescence quantum yield we made comparative measurements on UiO‐abdc and a crystal of Coumarin 120 under identical excitation and detection conditions, at ambient pressure (Figure S6). The fluorescence quantum yield of Coumarin 120 in the solid state is 0.01.^[21]^ The fluorescence of Coumarin 120 was detected easily, and 1 % of this intensity would be sufficient to be detectable (a signal‐to‐noise ratio of 3) under these conditions (Figure S7). Therefore, we estimate that the quantum yield of the azobenzene chromophore is <10^−4^.

This extremely low quantum yield seems surprising at first sight, given that photoisomerization cannot occur. However, this is consistent with the very low values reported for azobenzene in other highly constrained environments, as discussed above.[^11^, ^12^, ^13^] It is important to recall that efficient non‐radiative decay does not require attainment of the *cis* isomer equilibrium geometry, it only needs relaxation to a conformation that facilitates efficient internal conversion. Although the azobenzene is ostensibly constrained in the UiO framework, our previously reported crystal structure revealed dynamic disorder due to local twisting of the azo moiety. The conformational interconversion corresponds to a pedal‐like motion of the C−N=N−C group (Figure S8), and is the same as that first identified by Harada et al. in crystals of azobenzene and stilbene.^[22]^ This local conformational mobility will permit structural relaxation on the excited state potential energy surface, analogous to the proposed hula‐twist mechanism of photoisomerization, leading to fast internal conversion. The absence of fluorescence under pressure in FC‐70 suggests that bending of the linker does not inhibit the local motion of the azo group to an extent that significantly increases the quantum yield. We believe that the mediation of efficient non‐radiative decay by local twisting of the azo group offers a general explanation for the low fluorescence quantum yields exhibited by azobenzene in systems where photoisomerization is impeded, but this has not been recognised previously because of the lack of structural evidence.

## Conclusions

We have shown that use of the UiO framework as a platform for photophysical measurements offers a new perspective on the azobenzene chromophore. The ability to correlate spectroscopic and structural data has been particularly informative in elucidating the effects of pressure‐induced solvation, conformational distortion and local conformational mobility on photophysical properties. We anticipate that this approach will be particularly valuable in exploring excited‐state potential energy landscapes, by permitting access to crystallographically defined excited‐state geometries that lie outside the normally attainable Franck‐Condon region.

## Conflict of interest

The authors declare no conflict of interest.

## Supporting information

As a service to our authors and readers, this journal provides supporting information supplied by the authors. Such materials are peer reviewed and may be re‐organized for online delivery, but are not copy‐edited or typeset. Technical support issues arising from supporting information (other than missing files) should be addressed to the authors.

Supporting Information
