# Supplementary material for: Photophysics of Azobenzene Constrained in a UiO Metal–Organic Framework: Effects of Pressure, Solvation and Dynamic Disorder
Source: Chemistry. 2021 Oct 4;27(60):14871–5. doi: 10.1002/chem.202101879 (PMC8596631; doi:10.1002/chem.202101879)
Supplement: Supplementary file 1 — Supporting Information [file CHEM-27-14871-s001.pdf]

Chemistry–A European Journal

Supporting Information

Photophysics of Azobenzene Constrained in a UiO Metal–Organic Framework: Effects of Pressure, Solvation and Dynamic Disorder

Alif Sussardi, Ross J. Marshall, Stephen A. Moggach, Anita C. Jones,* and Ross S. Forgan*

Supporting Information

S1. Experimental Details

Sample preparation

Before measurement, the Zr-abdc MOF crystals were washed with acetone and left to dry at ambient temperature and pressure. Spectra were measured with either Fluorinert FC-70 or methanol as the hydrostatic media. In the case of FC-70, a crystal was loaded into the DAC alongside ruby, with clear Corning high-vacuum grease used as an adhesive for the crystal and the diamond culet. In the case of methanol, clear (Part A) Araldite® rapid resin was used as an adhesive for the crystal, instead of vacuum grease, due to solubility of the high-vacuum grease in methanol. The DAC was then placed in the custom-made measurement setup, as shown in Figure S1 (absorption spectra) and Figure S2 (fluorescence spectra).

Measurement of UV-vis absorption spectra

A broadband Ocean Optics balanced deuterium-halogen lamp (DH-2000-BAL) was used as the light source. The light was focussed into the DAC using an infinity-corrected 15x reflective-objective lens (Edmund Optics). The transmitted light from the DAC was collected by a second reflective-objective lens, and an image of the cell was projected onto a plane coincident with the optical fibre input of the Ocean Optics USB-2000+ spectrometer. The reference spectrum, I_0, and the transmitted spectrum of the sample, I_S, were recorded by carefully translating the DAC on a 3-axis MicroBlock compact flexure stage (THORLABS). In this way, an image of either an empty part of the cell or the sample crystal was directed onto the spectrometer input. The spectra were processed using SpectraSuite software (Ocean Optics).

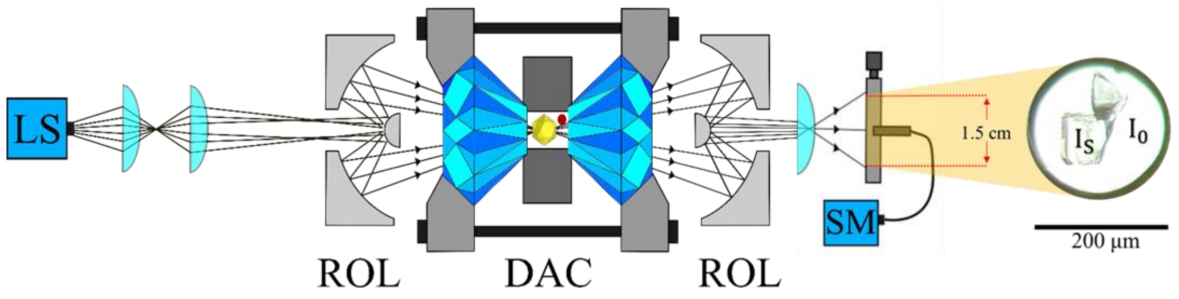

Figure S1. Schematic diagram of the experimental set-up for measurement of UV-vis absorption spectra as a function of pressure. LS: Light Source, ROL: reflective objective lens, DAC: diamond anvil cell, SM: spectrometer.

Measurement of fluorescence emission spectra

The experimental set-up for fluorescence measurements is shown schematically in Figure S2.

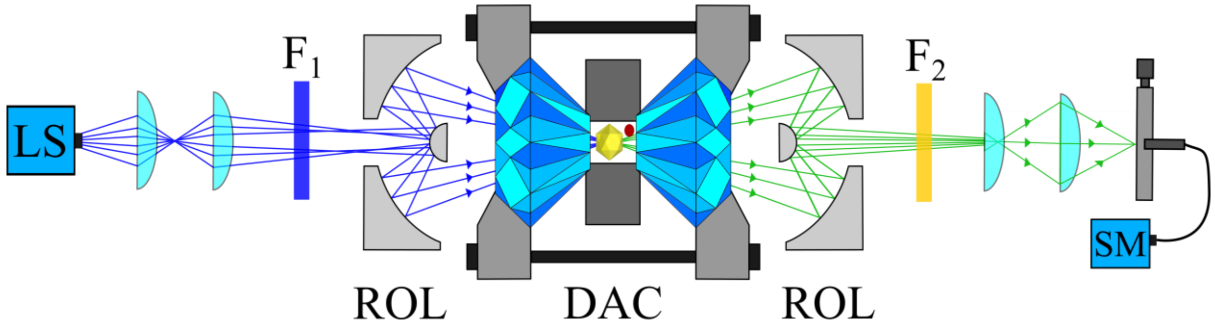

Figure S2. Schematic layout of experimental set-up for measurement of fluorescence emission spectra. LS: LED light Source, F1: band-pass filter; ROL: reflective objective lens, DAC: diamond anvil cell, F2: long-pass filter.

The set-up is similar to that used for measurement of absorption spectra, but differs in the excitation source and the collection optics. A 365-nm LED (Ocean Optics LS-LED) was utilised as the excitation source. Two plano-convex fused-silica lenses (f 30mm and 10mm) guided the excitation light into an infinity-corrected 15x reflective-objective lens (Edmund Optics) which focused the light into the DAC. The fluorescence from the sample in the DAC was collected using a second ROL and focused, using a second pair of fused silica lenses, into a fibre-coupled spectrometer (Ocean Optics USB2000+), controlled by SpectraSuite (Ocean Optics) software. A band-pass filter (Semrock Brightline 370/36) was inserted in the

excitation path to narrow the excitation bandwidth. A long-pass filter was inserted in the detection path to prevent the transmitted excitation light from reaching the detector. Fluorescence spectra were corrected for the wavelength-response of the spectrometer.

S2. Absorption Spectral Data

Table S1. Wavelength and wavenumber of the UV-vis absorption red-edge in Fluorinert FC-70 as a function of pressure.

**Pressure /GPa**	**Absorption Edge /nm**	**Absorption Edge /cm^{-1}**
ambient	544	18380
0.21	547	18280
0.3	548	18250
0.6	553	18080
0.82	553	18060
1.13	554	18040
1.84	555	18020
2.1	554	18050

Table S2. Wavelength and wavenumber of the UV-vis absorption red-edge in methanol as a function of pressure.

**Pressure /GPa**	**Absorption Edge /nm**	**Absorption Edge /cm^{-1}**
ambient	535	18680
0.25	534	18720
0.47	532	18800
0.63	532	18800
1.26	531	18820
1.49	531	18820
2.31	533	18750
3.28	535	18690
3.64	536	18640
4.09	538	18600

S3. DFT and TDDFT Calculations

Table S3. The values of the linker length, d_{CO}, used in the DFT calculations, and the corresponding pressures. The pressure values were determined from the relationship between d_{CO} and pressure shown in Figure S3.

d_{CO} / Å	Pressure / GPa
13.144	0.00
13.110	0.12
13.019	0.37
12.960	0.50
12.921	0.59
12.820	0.80
12.772	0.90
12.627	1.22
12.500	1.58
12.466	1.74

Table S4. The TDDFT-predicted wavelengths, λ, and oscillator strengths, *f*, of the S_1 nπ* and S_2 ππ* transitions, as a function of pressure, i.e for the vertical transitions from the DFT-optimised ground-state geometries, corresponding to the abdc ligand lengths determined from the unit cell parameters.

0.00 GPa		0.12 GPa		0.37 GPa		0.50 GPa		0.59 GPa	
λ /nm	*f*	λ /nm	*f*	λ /nm	*f*	λ /nm	*f*	λ /nm	*f*
417.0	0	416.2	0.001	418.1	0.009	419.4	0.014	420.4	0.017
318.4	1.024	319.2	1.016	319.4	0.990	319.5	0.975	319.5	0.966
**0.80 GPa**		**0.90 GPa**		**1.22 GPa**		**1.58 GPa**		**1.74 GPa**	
λ /nm	*f*	λ /nm	*f*	λ /nm	*f*	λ /nm	*f*	λ /nm	*f*
422.6	0.024	423.7	0.028	426.8	0.036	429.6	0.043	430.3	0.044
319.6	0.942	319.7	0.933	319.8	0.905	312.0	0.880	320.0	0.874

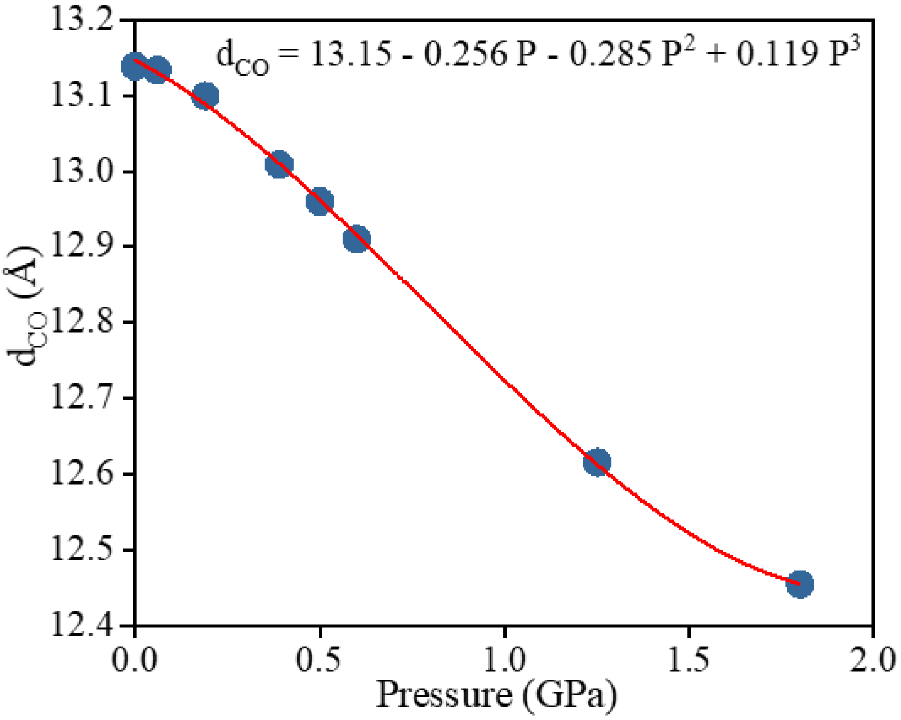

Figure S3. The dependence of linker length, d_{CO}, on pressure, determined from the unit cell parameters reported in reference S1. The relationship between d_{CO} and pressure can be fitted empirically by a third-order polynomial, as shown by the red curve.

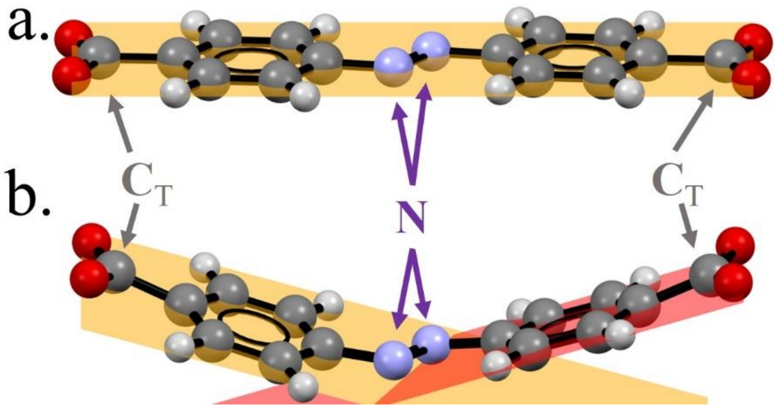

Figure S4. DFT-calculated structures corresponding to (a) ambient pressure (C_T-C_T distance 13.144 Å) and (b) 1.7 GPa (C_T-C_T distance 12.466 Å).

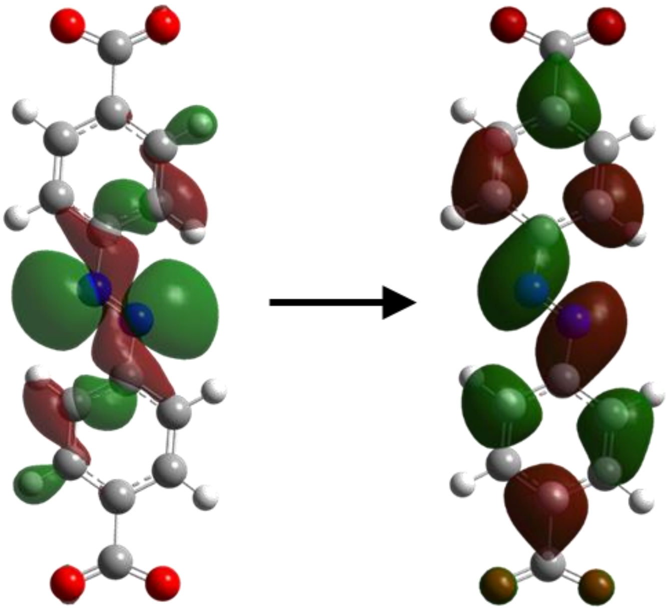

Figure S5. The orbitals involved in the lowest energy (~420 nm) electronic transition of azobenzene dicarboxylate.

S4. Estimation of Fluorescence Quantum Yield

Comparative measurements were made on UiO-abdc and Coumarin 120 (QY = 0.01), under identical conditions, at ambient pressure. The spectrum recorded for Coumarin 120 is shown in Figure S6, together with the signal recorded for UiO-abdc. The Coumarin 120 emission intensity is shown quantitatively in Figure S6, together with the noise on the baseline.

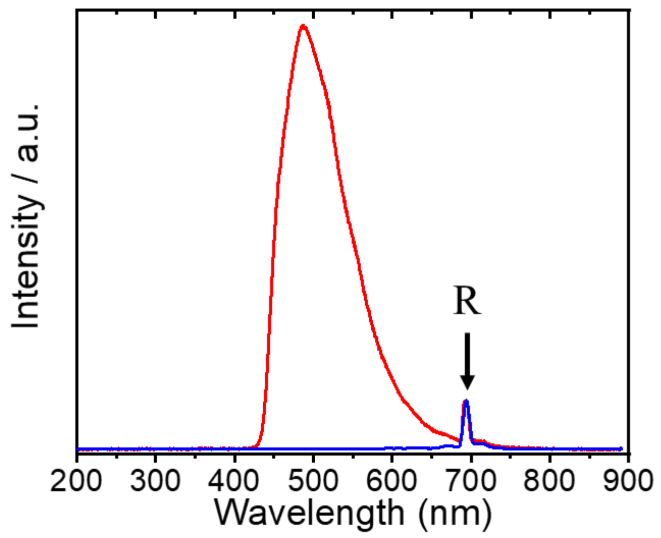

Figure S6. Fluorescence spectra of Coumarin 120 (red) and UiO-abdc (blue), recorded under the same excitation/detection conditions, with a signal integration time of 2 s. The spectra are normalised to the ruby emission peak (R) at ~695 nm.

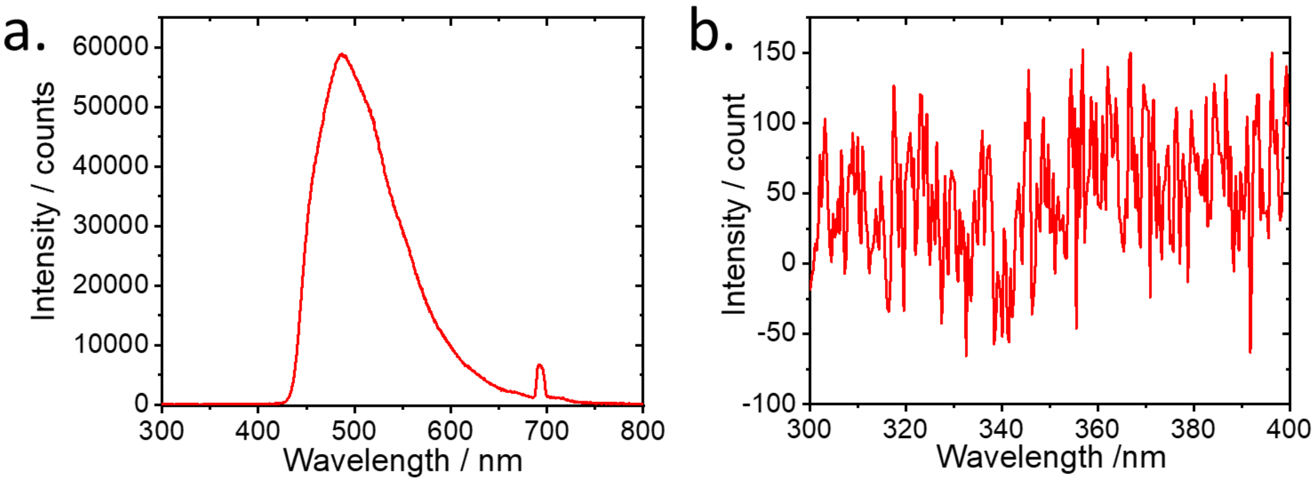

Figure S7. (a) Fluorescence spectrum of Coumarin 120 (absolute intensity). (b) The noise on the baseline.

S5. Dynamic Disorder in Crystal Structure

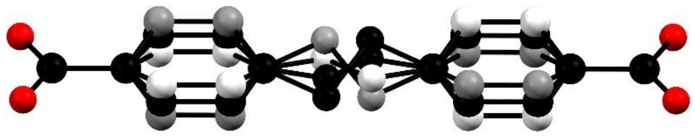

Figure S8. The pedal-like motion of the CN=NC group that is manifested as dynamic disorder in the crystal structure of UiO-abdc, as reported by Hobday *et al*.[S1]

[S1]. Hobday, C. L.; Marshall, R. J.; Murphie, C. F.; Sotelo, J.; Richards, T.; Allan, D. R.; Düren, T.; Coudert, F.-X.; Forgan, R. S.; Morrison, C. A.; Moggach, S. A.; Bennett, T. D., A Computational and Experimental Approach Linking Disorder, High-Pressure Behavior, and Mechanical Properties in UiO Frameworks. *Angewandte Chemie International Edition* **2016,** *55* (7), 2401-2405.
